# The Association between Anthropometric Failure and Toilet Types: A Cross-Sectional Study from India

**DOI:** 10.4269/ajtmh.22-0138

**Published:** 2023-02-13

**Authors:** Anoop Jain, Helen O. Pitchik, Caleb Harrison, Rockli Kim, S.V. Subramanian

**Affiliations:** ^1^ Department of Global Health & Social Medicine, Harvard Medical School, Boston, Massachusetts;; ^2^ Division of Epidemiology, School of Public Health, University of California, Berkeley, California;; ^3^ Department of Epidemiology, University of Michigan School of Public Health, Ann Arbor, Michigan;; ^4^ Division of Health Policy & Management, College of Health Sciences, Korea University, Seoul, South Korea;; ^5^ Department of Social and Behavioral Sciences, Harvard T.H. Chan School of Public Health, Boston, Massachusetts;; ^6^ Harvard Center for Population and Development Studies, Cambridge, Massachusetts

## Abstract

Sustainable Development Goal 6.2 aims to end open defecation by 2030 by ensuring universal access to private household toilets. However, private toilets might not be feasible for poor households. As a result, policy makers and academics have suggested well-managed shared sanitation facilities as an alternative solution. Less is known about the associations between shared sanitation use and health. Using data from the fifth round of the National Family Health Survey from 2019 to 2021, we estimated the association between usual defecation location and child anthropometry outcomes among children under 2 years in India. The primary exposure was usual defecation location at the household level. We compared both shared improved toilet use and open defecation to private, improved toilet use. We used linear regression to estimate the associations between the exposures and linear outcomes: height-for-age Z-score, weight-for-height Z-score, and weight-for-age Z-score. We used Poisson regression with a log link to estimate the prevalence ratios of stunting, wasting, and underweight. After controlling for environmental, maternal, socioeconomic, and child confounders, we found no differences in six child anthropometry outcomes when comparing shared toilet use or open defecation to private toilet use. This finding was consistent across both urban and rural households. Our findings confirm the null associations between private toilet use and child growth found in previous studies, and that this association does not vary if the toilet is being shared. Future research should examine these differences between private and shared toilets in the context of other health outcomes.

## INTRODUCTION

Since the mid-1980s, India’s government has led several major sanitation policies that aim to end open defecation by improving access to private household toilets. During this time, the prevalence of households reporting that they regularly defecate in the open decreased from 74% in 1990, to 50% in 2011.^1^ By 2020, the prevalence of households reporting that they regularly defecate in the open had decreased to 15%.^2^ Although this is a dramatic improvement, more than 200 million people continued defecating in the open every day throughout India as of 2020.^2^

Yet private toilets might not be feasible for many poor households, which could explain why open defecation remains an issue throughout India. More than a third of India’s 450 million urban residents live in densely populated informal dwellings and do not always have space for private toilets.^3^^,^^4^ Private toilets are also infeasible in some rural contexts, where many households cannot afford the upfront cost of toilet construction and do not have enough dwelling space.^5^^–^^7^ Poor soil quality and inadequate access to water for self-cleaning and flushing are also reasons why many households do not have a private toilet.^8^^–^^10^

Well-managed, shared toilets have been posited as a way to move people up the sanitation ladder away from open defecation in communities where households cannot build their own private toilet.^11^^–^^15^ Currently, however, all forms of shared sanitation are considered “limited” according to the WHO’s Joint Monitoring Program for Water Supply and Sanitation (JMP). This is true even if the shared toilet is improved (i.e., a flush/pour flush toilet connected to a piped sewer system, septic tank, or pit latrine or a pit latrine with a slab).^2^ A household has “safely managed” sanitation if it is using an private improved toilet in which the excreta is safely disposed of in situ or removed off site.^2^ Households using private improved toilets in which the method or location of excreta disposal is unknown have “basic” sanitation.^2^ Thus, the distinction between “limited” and “safely managed/basic” sanitation is based on whether more than one household is using an improved toilet, regardless of where the excreta are disposed.^2^ These definitions are presented in Table 1.

**Table 1:** JMP toilet typology

	JMP definition (ordered from highest to lowest quality)	Definition	Sharing status
Improved	Safely managed	Improved facilities that are not shared with other households and where excreta are safely disposed of in situ or removed and treated offsite	Not shared
	Basic	Improved facilities that are not shared with other households	Not shared
	Limited	Improved facility shared with other households	Shared
Unimproved	Unimproved	Use of pit latrines without a slab or platform, hanging latrines, or bucket latrines	Either
	Open defecation	Disposal of human feces in fields, forests, bushes, open bodies of water, beaches, or other open places	No facility

JMP = WHO Joint Monitoring Programme. Improved toilet facility: flush/pour flush toilets that are connected to a piped sewer system, septic tank, or pit latrine. Adapted from JMP progress report (2020).

The distinction between shared and private toilets is due to concerns that shared toilets are harder to maintain and unsanitary.^16^ Unsanitary conditions could expose users to fecal contamination, which is associated with a number of communicable diseases such as soil-transmitted helminth infections, trachoma, diarrhea, and schistosomiasis.^17^^,^^18^ In India, approximately 107,000 under-5 deaths, or 9% of the 1.2 million total under-5 deaths, were attributable to diarrhea in 2015.^19^ Additionally, the ingestion of fecal bacteria is associated with environmental enteric dysfunction and anthropometric failure including stunting, wasting, and underweight.^20^^–^^23^ More than 30% of the world’s stunted (< −2 SD height-for-age Z-score) children live in India.^24^ More than 15% of Indian children experience wasting (< −SD weight-for-height Z-score), and 32% of Indian children are underweight (< −SD weight-for-age Z-score).^25^ Exposure to fecal contamination might also increase susceptibility for acute respiratory infections.^26^^,^^27^

However, research has not consistently found that shared toilet quality is worse than that of private toilets. Cleanliness, privacy, lockable doors, availability of water (for flushing and self-cleaning), and proper construction have been identified as important indicators of quality by users of both private and shared toilets.^14^^,^^28^^,^^29^ One study showed that accessing water for flushing and self-cleaning is difficult even when households own private toilets.^30^ Users of household toilets built by the government in rural Bihar, India, cited poor quality as a deterrent to consistent use.^31^ Users of both private and shared toilets in Odisha, India, reported inadequate access to water, unsafe conditions, and uncleanliness.^32^

Further, the evidence regarding the extent to which the use of shared toilets is more harmful for health than private toilet use is mixed. A few studies have found that shared toilets are associated with poorer health. In one study, children in households that shared toilets with other households were at an increased risk for moderate-to-severe diarrhea compared with children using a private toilet in Kenya (odds ratio [OR]: 1.41, 95% CI: 1.11–1.79), Mali (OR: 1.23, 95% CI: 1.02–1.48), and Pakistan (OR: 1.58, 95% CI: 1.19–2.09).^33^ Similarly, use of shared toilets in India was associated with greater risk of norovirus infection (OR: 2.05, 95% CI: 1.09–3.86) compared with private toilet use, whereas shared toilet use in a Kenyan refugee camp was associated with an increased risk of watery diarrhea (OR: 2.17, 95% CI: 1.01–4.68) compared with private toilet use.^34^^,^^35^ In contrast, in some settings, shared toilet use is associated with a decreased risk of child diarrhea compared with private toilet use.^16^ In others, no difference has been found between outcomes across shared and private toilet use. This includes a study from rural Tanzania that found no difference in risk of trachoma between those who use private toilets versus shared and one from India that found no differences in levels of fecal contamination of household drinking water or household member hands between households using shared versus private toilets.^36^^,^^37^

Therefore, the primary aim of this work is to estimate the association between type of improved toilet use (shared versus private) and measures of child growth in India. Doing so is important given calls for well-managed, shared sanitation in communities where households are unable to build or use a private toilet.^11^^–^^15^ These findings will help inform whether the distinction between “limited” and “safely managed” sanitation matters in the context child growth.

## MATERIALS AND METHODS

### Data source.

We used the fifth round of India’s National Family Health Survey (NFHS-5) dataset which was collected between 2019 and 2021. Households were selected through two-stage sampling. Primary sampling units (PSUs) were villages in rural areas and wards in urban areas and were selected with probability proportional to size sampling.^38^ Households were then selected from PSUs in the second stage.^39^ We studied children under age 2 years. The first 2 years of life are a critical period for growth,^40^ which is why previous sanitation and health studies have also focused on children of this age.^41^^–^^43^ The NFHS-5 contains data from all 36 Indian states/union territories, 707 districts, and 30,198 PSUs.

### Outcome and exposure definitions.

We included six child anthropometry outcomes as a part of this study. These included three linear variables: height-for-age Z-score (HAZ), weight-for-height Z-score (WHZ), and weight-for-age Z-score (WAZ), and three binary variables: stunting (< −2 SD HAZ), wasting (< −2 SD WHZ), and underweight (< −2 SD WAZ) status, binary variables defined according to WHO standards.^32^ We assessed the association between sanitation and child growth because current evidence is mixed. Some studies show that inadequate access to sanitation is associated with poor child growth,^44^^–^^46^ whereas others show no effect.^42^^,^^43^^,^^47^^,^^48^ We did not include child diarrhea because this outcome is reported retrospectively in our data source.

The exposure was toilet type. As a part of the survey, respondents were first asked what kind of toilet facility the members of the household have access to and were then asked if this facility was shared with other households. The exposure was grouped into three categories: open defecation, private improved toilet facility, and shared improved toilet facility. Private improved toilet was the reference group for all analyses, allowing us to compare outcomes between 1) private toilets and open defecation and 2) private toilets and shared toilets. We excluded households that relied on unimproved toilets given that these types of toilets do not hygienically separate human excreta from human contact.^2^

### Covariates.

We adjusted for theoretical confounders of the association between toilet type and child anthropometry.^49^^–^^51^ These are shown in a supplemental directed acyclic graph (Supplemental Figure 1) and were split in to four main categories: environmental, maternal, socioeconomic, and child characteristics. In the environmental category, we included household use of clean cooking fuel, animal ownership, and household access to improved drinking water, each of which was dichotomized as yes/no. In the maternal category, we included mother’s age at marriage, dichotomized above or below 18, whether a skilled birth attendant was present at birth, and mother’s education (categorized into no schooling, primary, secondary, and higher education), and mother’s body mass index (kg/m^2^) as a continuous variable. In the socioeconomic category, we included household wealth quintile, location, and household caste as indicators of socioeconomic status. Household location was either urban or rural, and household caste was either Scheduled Caste/Tribe, Other Backward Class, or General Caste. Additionally, in the child category, we controlled for birthweight (above or below 2.5 kg), child’s age in months, their birth order, and early breastfeeding initiation.

### Statistical analysis.

We conducted three analyses for each outcome. The first compared outcomes for all children who lived in household who usually use private improved toilets to those who use 1) shared improved toilets and 2) open defecation. Second, we stratified shared toilets by the number of households sharing the facility (two, three/four, and five or more based on the distribution in the sample of those sharing toilets) and compared outcomes for children in each group to those in households that usually use private improved toilets. Third, we stratified the sample by urban or rural location, and repeated analyses 1 and 2 in each stratum. Analyses 1 and 2 included both unadjusted and adjusted models. Analysis 3 was only done with adjusted models. We used linear regression to estimate the mean difference in continuous HAZ, WHZ, and WAZ outcomes and Poisson regression with a log link and robust standard errors to estimate the prevalence ratios for binary child stunting, wasting, and underweight outcomes.^52^^,^^53^ For all outcomes, we clustered the standard errors at the PSU level to account for the sampling design.

## RESULTS

### Overall sample characteristics.

Our final sample included children under 2 years with complete exposure, outcome, and confounder data (*N* = 60,949). Descriptive statistics for each covariate stratified by exposure group are presented in Table 2. When comparing the children who were excluded due to missing data on exposure, outcome, or confounders (*N* = 25,200) to those who were included in the analysis, the characteristics were similar on multiple demographic characteristics. The children in the analytic sample were less likely to be in the lowest wealth quintile, less likely to have a mother with no formal education, and less likely to have been born with a skilled birth attendant present compared with the children excluded because of missing data (Supplemental Table 1). A total of 13,590 children (22%) lived in households where family members usually practiced open defecation. An additional 42,076 children (69%) lived in households with a private toilet, and 5,283 children (9%) lived in households with a shared toilet. Descriptive statistics for the analytic sample are presented in Table 2, and the number of children by type of toilet are presented in Table 3.

**Table 2:** Descriptive statistics of variables the analytic sample

Variables			Private		Shared		Open Defecation	
			*n*	% of category	*n*	% of category	*n*	% of category
Environ. variables	Cooking fuel source	Solid fuel	19,487	46	2,445	46	10,685	79
		Clean fuel	22,589	54	2,838	54	2,905	21
		Total	42,076		5,283		13,590	
	Drinking water source	Unimproved	3,993	9	554	10	1,453	11
		Improved	38,083	9	4,729	90	12,137	89
		Total	42,076		5,283		13,590	
	Animal ownership	Has animal	23,186	55	2,262	43	8,912	66
		No animal	18,890	45	3,021	57	4,678	34
		Total	42,076		5,283		13,590	
Maternal variables	Maternal marriage	< 18	9,853	23	1,542	29	5,349	39
		18+	32,223	77	3,741	71	8,241	61
		Total	42,076		5,283		13,590	
	Skilled birth attendant	No	2,681	6	359	7	1,264	9
		Yes	39,395	94	4,924	93	12,326	9
		Total	42,076		5,283		13,590	
	Maternal education	No schooling	5,205	12	862	16	4,747	35
		Primary	4,154	10	709	13	2,110	16
		Secondary	24,013	57	3,124	59	6,102	45
		Higher	8,704	21	588	11	631	5
		Total	42,076		5,283		13,590	
SE variables	Household wealth quintile	Poorest	5,847	14	890	17	7,672	56
		Poor	8,340	20	1,492	28	4,008	29
		Middle	9,447	22	1,545	29	1,596	12
		High	9,902	24	1,014	19	301	2
		Highest	8,540	20	342	6	13	0
		Total	42,076		5,283		13,590	
	Household location	Rural	32,055	76	3,822	72	12,739	94
		Urban	10,021	24	1,461	28	851	6
		Total	42,076		5,283		13,590	
	Household caste	Scheduled Caste	8,240	20	1,323	25	3,562	26
		Scheduled Tribe	8,412	20	759	14	3,376	25
		Other Backward Class	17,089	41	2,135	40	5,544	41
		Other	8,335	20	1,066	20	1,108	8
		Total	42,076		5,283		13,590	
Child variables	Child birthweight	< 2.5 kg	6,828	16	970	18	2,778	20
		> 2.5 kg	35,248	84	4,313	82	10,812	80
		Total	42,076		5,283		13,590	
	Breastfeeding initiation	>1 hour	22,727	54	2,920	55	7,792	57
		< 1 hour	19,349	46	2,363	45	5,798	43
		Total	42,076		5,283		13,590	
	Child birth order	First	17,557	42	1,932	37	4,431	33
		Second/third	20,335	48	2,782	53	6,778	50
		Fourth/fifth	3,323	8	481	9	1,906	14
		Sixth +	861	2	88	2	476	4
		Total	42,076		5,283		13,591	
			M	SD	M	SD	M	SD
Continuous variables	Child age (months)		12.3	7.01	12.8	6.96	12.4	7.05
	Mother’s BMI		21.96	4.04	21.55	3.87	20.3	3.23

BMI = body mass index; Environ. = environmental; SE = socioeconomic.

**Table 3:** Number of children by usual household defecation location overall and by household location

	All India	Rural	Urban
Open defecation	13,590	12,739	851
Private	42,076	32,055	10,021
Any sharing	5,283	3,822	1,461
Two households	3,187	2,512	675
Three/four households	1,470	1,013	457
Five or more households	626	297	329
Total	60,949	48,616	12,333

The mean HAZ score in our sample was −1.13 (SD 1.97). The mean WHZ and WAZ scores were −0.77 (SD 1.64) and −1.22 (SD 1.31), respectively. In our sample, 23,374 children (32%) were stunted, 15,163 (21%) were wasted, and 18,812 (26%) were underweight. The mean values for HAZ, WHZ, and WAZ, and the prevalence of each binary anthropometry outcome in each of the three models by toilet type are presented in Supplemental Table 2.

### Open defecation.

**Figure 1.**
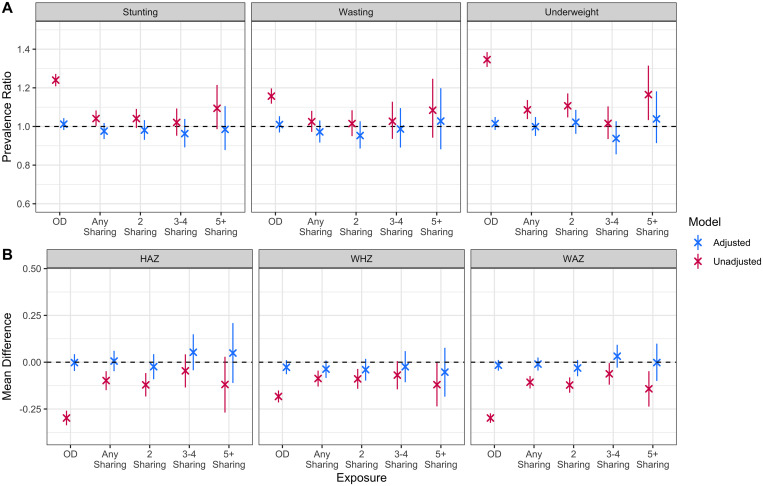
Adjusted and unadjusted prevalence ratios for binary outcomes and mean difference for continuous outcomes for the relationship between toilet-type and anthropometric outcomes. Private toilet use is the reference category for all analysis. “2 sharing”, “3/4 sharing”, and “5+ sharing” are all subgroups of the “any sharing” category and were analyzed in separate models. HAZ = height-for-age Z-score; WAZ = weight-for-age Z-score; WHZ = weight-for-height Z-score.

In our unadjusted analyses, open defecation was associated with lower mean HAZ (−0.30, 95% CI: −0.34 to −0.26), WHZ (−0.18, 95% CI: −0.22 to −0.15), and WAZ (−0.30, 95% CI: −0.32 to −0.27) compared with private toilet use. After adjusting for potential confounders of the association between private toilet access and child anthropometry, the mean differences were smaller in magnitude: −0.002 for HAZ (95% CI: −0.05 to 0.04), −0.03 for WHZ (95% CI: −0.06 to 0.01), and −0.02 for WAZ (95% CI: −0.05 to 0.01). In unadjusted models, open defecation was associated with a higher risk of child stunting (relative risk [RR]: 1.24, 95% CI: 1.21–1.27), wasting (RR: 1.16, 95% CI: 1.12–1.20), and underweight (1.35, 95% CI: 1.31–1.39) compared with private toilet use. After adjusting for potential confounders of the association between open defecation and child anthropometric failure, these prevalence ratios were smaller in magnitude: 1.01 for stunting (95% CI: 0.98–1.04), 1.01 for wasting (95% CI: 0.97–1.05), and 1.02 for underweight (95% CI: 0.98–1.05). These results are presented in Figure 1 and Supplemental Table 3.

### Shared toilet use.

Shared toilet use was associated with lower mean HAZ (−0.10, 95% CI: −0.15 to −0.05), WHZ (−0.09, 95% CI: −0.13 to −0.05), and WAZ (−0.11, 95% CI: −0.14 to −0.07) compared with private toilet use in unadjusted models. After adjusting for potential confounders of the association between toilet type and child anthropometry, the mean differences were attenuated: 0.006 for HAZ (95% CI: −0.05 to 0.06), −0.04 for WHZ (95% CI: −0.08 to 0.01), and −0.01 for WAZ (95% CI: −0.05 to 0.03). The unadjusted prevalence ratios for child stunting, wasting, and underweight for children using shared toilets were 1.04 (95% CI: 1.00–1.08), 1.03 (95% CI: 0.97–1.08), and 1.09 (95% CI: 1.04–1.14), respectively, compared with private toilet use. After adjusting for potential confounders between toilet type and child anthropometric failure, these prevalence ratios were smaller in magnitude: 0.98 for stunting (95% CI: 0.94–1.02), 0.97 for wasting (95% CI: 0.92–1.03), and 1.00 for underweight (95% CI: 0.95–1.05). These results are presented in Figure 1 and Supplemental Table 3.

When stratifying by the number of households sharing a toilet, the adjusted associations between shared toilet use and HAZ were between −0.02 (two sharing) and 0.05 (five or more sharing) when compared with private toilet use. For WHZ the adjusted mean differences were between −0.02 (three/four sharing) and −0.05 (five or more sharing) compared with private toilet use. For WAZ, the adjusted mean differences were between −0.03 (two sharing) and 0.03 (three/four sharing) when compared with private toilet use. After stratifying by the number of households sharing a toilet, the adjusted prevalence ratios for the relationship between shared toilet access and stunting ranged from 0.96 (three/four sharing) to 0.98 (two sharing and five or more sharing), 0.95 (two sharing) to 1.03 (five or more sharing) for wasting, and 0.94 (three/four sharing) to 1.04 (five or more sharing) for underweight, all when compared with open defecation. These results are presented in Figure 1 and Supplemental Table 4.

### Urban and rural analyses.

**Figure 2.**
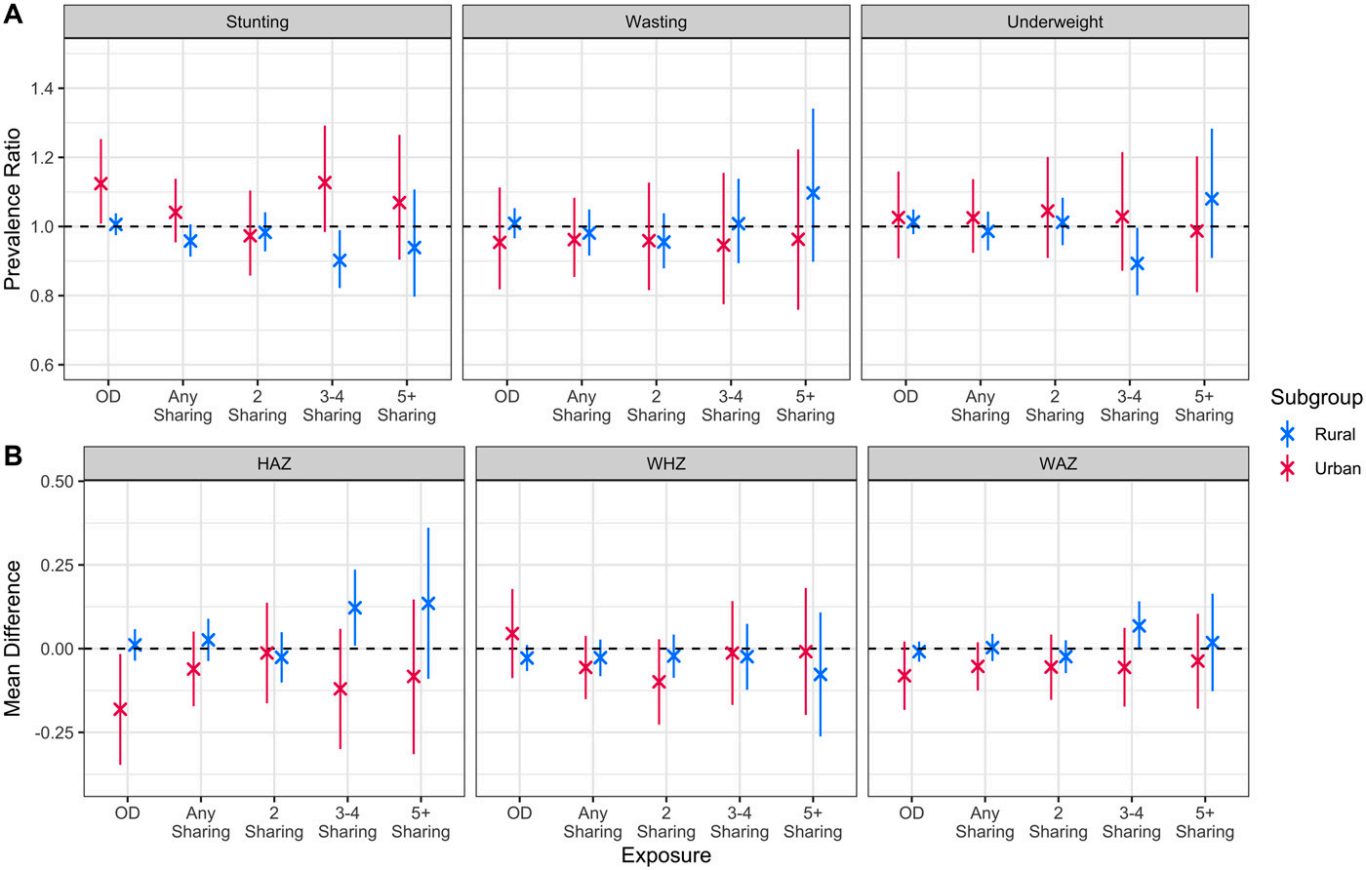
Adjusted prevalence ratios for binary outcomes and mean difference for continuous outcomes for the relationship between toilet-type and anthropometric outcomes within strata of household location (urban versus rural). Private toilet use is the reference category for all analyses. “2 sharing”, “3/4 sharing”, and “5+ sharing” are all subgroups of the “any sharing” category and were analyzed in separate models. HAZ = height-for-age Z-score; WAZ = weight-for-age Z-score; WHZ = weight-for-height Z-score.

Among children living rural households, open defecation was not associated with statistically significantly (*P* < 0.05) worse HAZ, WHZ, WAZ, stunting, wasting, or underweight outcomes compared with private toilet use. However, open defecation in urban settings was associated with significantly lower HAZ scores (−0.18, 95% CI −0.35 to −0.02) and a greater risk of stunting (1.12, 95% CI: 1.01–1.25) when compared with private toilet use. Among children living rural or urban households, shared toilet use was not associated with any statistically significant differences in child HAZ, WHZ, WAZ, stunting, wasting, or underweight compared with private toilet use. These results are presented in Figure 2 and Supplemental Tables 5 and 6.

Children in rural households using a toilet shared by three/four households had statistically significantly higher HAZ scores (0.12, 95% CI: 0.01–0.24) and were at a lower risk of stunting (0.90, 95% CI: 0.82–0.99) and underweight (0.89, 95% CI: 0.80–0.99) compared with children using private toilets. These were the only statistically significant relationships found when comparing rural children in households sharing a toilet with two, three/four, or five or more other households to those who use a private toilet. There were no statistically significant relationships found when doing the same comparisons with urban children. These results are presented in Figure 2 and Supplemental Tables 7 and 8.

## DISCUSSION

This study had two salient findings. First, after adjusting for theoretical confounders of the association between toilet type and child anthropometry, we found that six child growth outcomes (child HAZ, WHZ, WAZ, stunting, wasting, or underweight) did not differ for children living in households that used shared toilets compared with those that used private toilets. The same was true when comparing open defecation with private toilet use. Second, shared toilet use was not associated with any statistically significant differences in the same six growth outcomes compared with private toilet among urban or rural households. The only exception to this was that children in rural households using a toilet shared by three/four households had higher HAZ scores and were at a lower risk of stunting and underweight compared with children using private toilets. Similarly, open defecation was not associated with statistically significantly worse growth outcomes compared with private toilet use in rural or urban areas in our adjusted models, with one exception. Open defecation was associated with lower HAZ scores and a higher risk of stunting compared with private toilet use among urban households.

There are three data limitations with this study. First, a subset of the data was missing data on exposures, outcomes, or covariates used in the analysis. However, the characteristics of missing population were similar to those in the analytic sample, with differences not likely to induce strong bias, as shown in Supplemental Table 1. Second, although NFHS data are considered high quality,^54^ questions about some of the covariates included in this study were self-reported and not verified by enumerators, which introduces a potential source of measurement error due to social desirability bias. Outcomes were measured by enumerators, reducing the impact social desirability bias in this study. Third, NHFS data does not differentiate between “basic” toilet and “safely managed” toilet as defined by the JMP.^2^ A portion of the households have access to a private toilet considered “safely managed,” which are of higher quality. We did not isolate this difference when comparing open defecation and shared toilets to private toilets.

Our results show that the relationship between sanitation and child growth does not vary by toilet-sharing status. Further, similar to previous findings, our results show no difference when comparing private toilet use to open defecation in terms of child growth outcomes. Previous work has shown that private toilets can be protective against moderate-to-severe diarrhea in rural Kenya.^33^ Our findings show that neither private nor shared toilets were protective against growth faltering regardless of household location. In a cluster randomized controlled trial assessing the effects of water, sanitation, hygiene, and nutrition on diarrhea and child growth in rural Bangladesh, no significant differences were found in length-for-age Z-scores among children in the sanitation only arm (−0.02, 95% CI: −0.14 to 0.09) compared with children in the control group.^55^ No significant differences were detected for weight-for-age (−0.00, 95% CI: −0.11 to 0.11) or weight-for-height (0.01, 95% CI: −0.09 to 0.11) Z-scores in this trial.^55^ In a similar cluster-randomized trial in Zimbabwe, no significant improvement in length-for-age Z-score were found among children who had sanitation (0.06, 95% CI: −0.01 to 0.12) compared with children without sanitation.^56^ Two studies similarly found that increased household toilet coverage is not sufficient to improve child growth in India.^47^^,^^48^ Despite high rates of toilet adoption and behavior change, the prevalence of enteropathogens among children in these trials was significantly higher compared with children in wealthier nations.^57^

These two findings are important for several reasons. First, child growth outcomes do not appear to be tied to toilet-sharing status in the Indian context regardless of household location. This could be because children might be exposed to many of the same risk factors for poor growth in both urban and rural communities.^58^ Second, household-level sanitation interventions might not be sufficient to improve child growth if not coupled with broader efforts to reduce fecal contamination in household environments.^57^ This is particularly important in rural areas where both agriculture and ground-water sources remain heavily polluted with fecal contamination.^59^^,^^60^ Additionally, factors such as healthcare coverage and household assets, such as refrigeration and clean cooking fuel, are all associated with improved child growth.^44^^,^^46^^,^^49^^,^^61^ Poor maternal nutrition, inadequate dietary diversity, and intrastate inequality are also associated with poor child linear growth.^62^^–^^67^ A few studies have shown positive associations between private toilet ownership and use and improved child HAZ.^68^^–^^70^ In these examples, sanitation interventions with longer durations, and interventions combined with access to other services, such as piped water to the household and improved nutrition, may have helped promote child growth. The importance of intervention fidelity for improvements in health outcomes has also been documented in the context of shared toilets.^71^ Thus, use of a toilet, be it private or shared, is just one of many potential determinants of child growth.^72^^–^^74^

Regardless of sharing status, toilets must be well-maintained and acceptable to users to encourage consistent use and prevent the spread of disease, a fact highlighted by the current global COVID-19 pandemic and worsening effects of climate change.^13^^,^^75^^–^^81^ Inadequate access to safe toilets is associated with poor psychosocial outcomes among women and girls.^32^^,^^82^^,^^83^ Therefore, ensuring access to safe and well-maintained toilet facilities, shared or private, could help improve these outcomes.^84^^,^^85^ In various contexts shared toilets have been found to be acceptable by users.^75^^,^^86^ Acceptability depends on factors such as availability of water at the facility, cleanliness, handwashing stations, gender-separated entrances, doors that lock, lighting for nighttime use, the presence of service staff, and the number of households sharing the toilet.^15^^,^^87^^,^^88^ Community-based approaches to promoting cleanliness at shared sanitation facilities could guide how these facilities are managed and financially sustained, which could influence user acceptability.^89^^–^^91^ Additionally, although shared sanitation has been thought of as most viable in dense urban communities, there is mounting evidence about the important role these facilities could play in both rural and periurban communities in the Indian context.^5^^,^^6^^,^^88^ Future research should examine the viability of shared sanitation in rural areas given that many of these communities also have high population densities, and this is where the burden of open defecation is the highest.^2^^,^^92^

In conclusion, sharing status is a major distinction used to assess toilet quality. The purpose of this study was to elucidate the extent to which the sharing status of a toilet matters for child anthropometry and anthropometric failure in India. We found no difference between shared toilet use and private toilet use with regard to child growth outcomes after adjusting for potential confounders of these associations. As policy makers and academics suggest that well-managed shared sanitation could help end open defecation in communities where private household toilets are infeasible, we show that in the Indian context this distinction is not related to child growth. Future research should examine the associations between shared toilet use and other health outcomes to further understand the implications of this infrastructure.

## Supplemental files


Supplemental materials
