# Supplementary material for: The Association between Anthropometric Failure and Toilet Types: A Cross-Sectional Study from India
Source: Am J Trop Med Hyg. 2023 Feb 13;108(4):811–9. doi: 10.4269/ajtmh.22-0138 (PMC10077020; doi:10.4269/ajtmh.22-0138)
Supplement: Supplementary file 1 [file tpmd220138.SD1.pdf]

Supplementary Figure 1: Directed Acyclic Graph of Child Growth

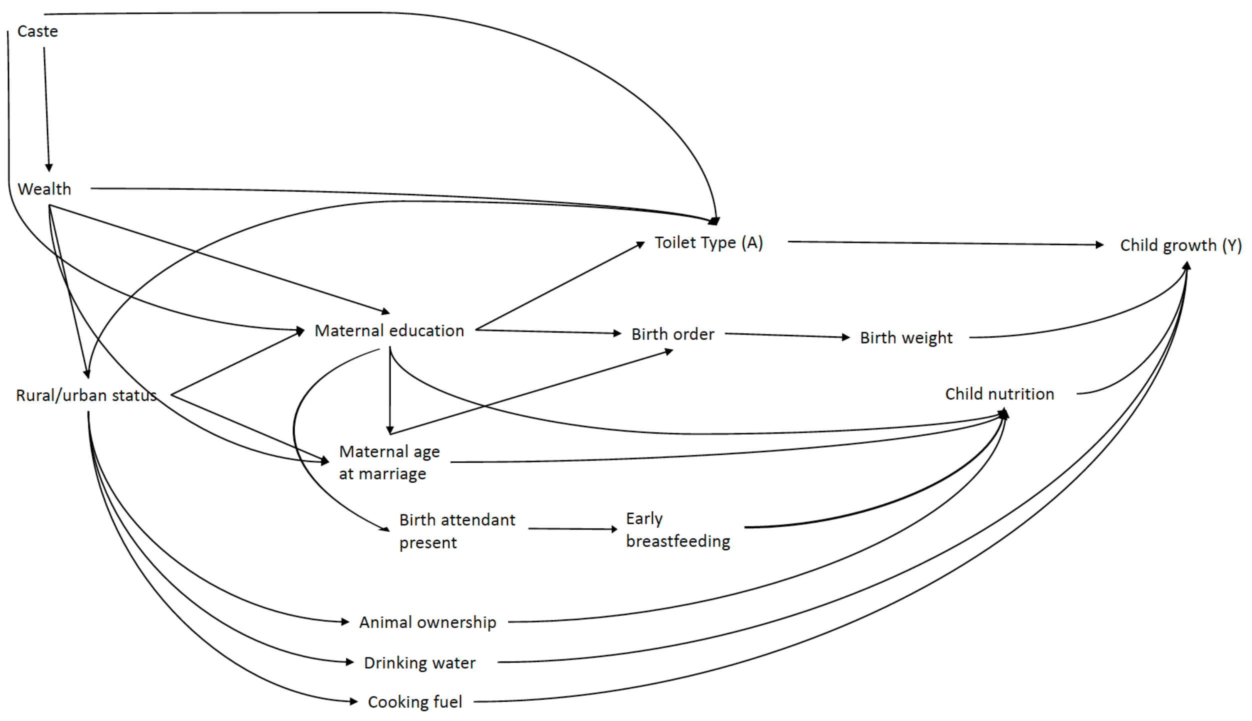

Supplementary Table 1: Missingness of observations by covariates

		Sample Analyzed		Missing	
		**N**	**% of Category**	**N**	**% of Category**
**Cooking Fuel Source**	Solid	32,617	54%	14,004	56%
	Clean	28,332	46%	11,187	44%
**Drinking water source**	Unimproved	6,000	10%	2,558	10%
	Improved	54,949	90%	22,642	90%
**Animal Ownership**	Has Animal	34,360	56%	14,627	58%
	No Animal	26,589	44%	10,573	42%
**Maternal marriage**	< 18	16,744	27%	7,419	29%
	18 +	44,205	73%	17,781	71%
**Birth Order**	First	23,290	39%	9,760	39%
	Second/Third	29,895	49%	11,863	47%
	Fourth/Fifth	5,709	9%	2,736	11%
	Sixth +	1,425	3%	841	3%
**Skilled birth attendant**	No	4,304	7%	5,256	21%
	Yes	56,645	93%	19,944	79%
**Maternal Education**	No schooling	10,814	18%	5,726	23%
	Primary	6,973	11%	2,990	12%
	Secondary	33,239	55%	12,603	50%
	Higher	9,923	16%	3,881	15%
**Household Wealth Quintile**	Poorest	14,409	24%	7,279	29%
	Poor	13,840	23%	5,759	23%
	Middle	12,588	21%	4,660	18%
	High	11,217	18%	4,090	16%
	Highest	8,895	15%	3,412	14%
**Household Location**	Rural	48,616	80%	20,002	79%
	Urban	12,333	20%	5,198	21%

Supplementary Table 2: Mean values for HAZ, WHZ, and WAZ, and the prevalence of each binary anthropometry outcome in each of the three analyses by toilet type. HAZ: height-for-age-Z-score. WHZ: weight-for-height-Z-score. WAZ: weight-for-age-Z-score.

	All India			Rural			Urban		
	**HAZ**	**WHZ**	**WAZ**	**HAZ**	**WHZ**	**WAZ**	**HAZ**	**WHZ**	**WAZ**
**No Sanitation**	-1.42	-1.03	-1.56	-1.41	-1.04	-1.56	-1.46	-0.93	-1.52
**Private Sanitation**	-1.04	-0.69	-1.11	-1.09	-0.71	-1.15	-0.86	-0.65	-0.97
**Shared Sanitation**	-1.18	-0.78	-1.23	-1.20	-0.77	-1.23	-1.10	-0.81	-1.20
	**Stunting**	**Wasting**	**Underweight**	**Stunting**	**Wasting**	**Underweight**	**Stunting**	**Wasting**	**Underweight**
**No Sanitation**	39%	25%	35%	39%	26%	35%	40%	24%	35%
**Private Sanitation**	30%	19%	23%	32%	20%	24%	26%	19%	20%
**Shared Sanitation**	32%	20%	25%	35%	20%	25%	31%	20%	25%

Supplementary Table 3: Growth Estimates (Comparing OD & Shared to Private Toilet Users). HAZ: height-for-age-Z-score. WHZ: weight-for-height-Z-score. WAZ: weight-for-age-Z-score. OD: open defecation. MD: mean difference. PR: prevalence ratio.

Exposure	Outcome	Model	Point Estimate	CI Lower	CI Upper	Measure of Association	Subgroup
OD	HAZ	Unadjusted	-0.298	-0.337	-0.259	MD	None
OD	HAZ	Adjusted	-0.002	-0.047	0.043	MD	None
OD	WHZ	Unadjusted	-0.183	-0.215	-0.151	MD	None
OD	WHZ	Adjusted	-0.027	-0.064	0.011	MD	None
OD	WAZ	Unadjusted	-0.298	-0.324	-0.273	MD	None
OD	WAZ	Adjusted	-0.016	-0.045	0.012	MD	None
OD	Stunting	Unadjusted	1.240	1.208	1.272	PR	None
OD	Stunting	Adjusted	1.012	0.982	1.043	PR	None
OD	Wasting	Unadjusted	1.157	1.118	1.197	PR	None
OD	Wasting	Adjusted	1.010	0.969	1.053	PR	None
OD	Underweight	Unadjusted	1.346	1.308	1.385	PR	None
OD	Underweight	Adjusted	1.015	0.982	1.049	PR	None
Shared	HAZ	Unadjusted	-0.098	-0.149	-0.048	MD	None
Shared	HAZ	Adjusted	0.006	-0.048	0.061	MD	None
Shared	WHZ	Unadjusted	-0.087	-0.130	-0.045	MD	None
Shared	WHZ	Adjusted	-0.037	-0.084	0.009	MD	None
Shared	WAZ	Unadjusted	-0.107	-0.140	-0.074	MD	None
Shared	WAZ	Adjusted	-0.010	-0.045	0.025	MD	None
Shared	Stunting	Unadjusted	1.041	1.003	1.083	PR	None
Shared	Stunting	Adjusted	0.976	0.935	1.018	PR	None
Shared	Wasting	Unadjusted	1.025	0.972	1.081	PR	None
Shared	Wasting	Adjusted	0.972	0.917	1.031	PR	None
Shared	Underweight	Unadjusted	1.086	1.038	1.137	PR	None
Shared	Underweight	Adjusted	0.999	0.951	1.049	PR	None

Supplementary Table 4: Shared Sanitation Estimates, Stratified by Number of Households Sharing. HAZ: height-for-age-Z-score. WHZ: weight-for-height-Z-score. WAZ: weight-for-age-Z-score. OD: open defecation. MD: mean difference. PR: prevalence ratio. "2 sharing", "3/4 sharing", and "5+ sharing" are all subgroups of the "any sharing" category and were analyzed in separate models.

Exposure	Outcome	Model	Point Estimate	CI Lower	CI Upper	Measure of Association	Subgroup
Shared	HAZ	Unadjusted	-0.121	-0.183	-0.058	MD	2 Sharing
Shared	HAZ	Adjusted	-0.024	-0.091	0.043	MD	2 Sharing
Shared	WHZ	Unadjusted	-0.089	-0.142	-0.036	MD	2 Sharing
Shared	WHZ	Adjusted	-0.040	-0.098	0.018	MD	2 Sharing
Shared	WAZ	Unadjusted	-0.122	-0.163	-0.081	MD	2 Sharing
Shared	WAZ	Adjusted	-0.031	-0.075	0.012	MD	2 Sharing
Shared	Stunting	Unadjusted	1.041	0.992	1.091	PR	2 Sharing
Shared	Stunting	Adjusted	0.981	0.931	1.033	PR	2 Sharing
Shared	Wasting	Unadjusted	1.015	0.950	1.084	PR	2 Sharing
Shared	Wasting	Adjusted	0.953	0.886	1.027	PR	2 Sharing
Shared	Underweight	Unadjusted	1.107	1.047	1.171	PR	2 Sharing
Shared	Underweight	Adjusted	1.022	0.962	1.086	PR	2 Sharing
Shared	HAZ	Unadjusted	-0.046	-0.135	0.042	MD	3/4 sharing
Shared	HAZ	Adjusted	0.053	-0.043	0.149	MD	3/4 sharing
Shared	WHZ	Unadjusted	-0.069	-0.145	0.006	MD	3/4 sharing
Shared	WHZ	Adjusted	-0.024	-0.107	0.059	MD	3/4 sharing
Shared	WAZ	Unadjusted	-0.062	-0.120	-0.005	MD	3/4 sharing
Shared	WAZ	Adjusted	0.032	-0.029	0.093	MD	3/4 sharing
Shared	Stunting	Unadjusted	1.021	0.952	1.093	PR	3/4 sharing
Shared	Stunting	Adjusted	0.963	0.892	1.039	PR	3/4 sharing
Shared	Wasting	Unadjusted	1.027	0.936	1.128	PR	3/4 sharing
Shared	Wasting	Adjusted	0.987	0.891	1.095	PR	3/4 sharing
Shared	Underweight	Unadjusted	1.016	0.935	1.104	PR	3/4 sharing
Shared	Underweight	Adjusted	0.938	0.856	1.027	PR	3/4 sharing
Shared	HAZ	Unadjusted	-0.119	-0.269	0.029	MD	5+ sharing
Shared	HAZ	Adjusted	0.049	-0.111	0.209	MD	5+ sharing
Shared	WHZ	Unadjusted	-0.120	-0.236	-0.003	MD	5+ sharing
Shared	WHZ	Adjusted	-0.053	-0.184	0.077	MD	5+ sharing
Shared	WAZ	Unadjusted	-0.142	-0.237	-0.048	MD	5+ sharing
Shared	WAZ	Adjusted	-0.002	-0.100	0.099	MD	5+ sharing
Shared	Stunting	Unadjusted	1.094	0.986	1.215	PR	5+ sharing
Shared	Stunting	Adjusted	0.985	0.878	1.105	PR	5+ sharing
Shared	Wasting	Unadjusted	1.084	0.942	1.247	PR	5+ sharing
Shared	Wasting	Adjusted	1.028	0.882	1.198	PR	5+ sharing
Shared	Underweight	Unadjusted	1.165	1.033	1.315	PR	5+ sharing
Shared	Underweight	Adjusted	1.039	0.914	1.182	PR	5+ sharing

Supplementary Table 5: Full Models for Rural Populations. HAZ: height-for-age-Z-score. WHZ: weight-for-height-Z-score. WAZ: weight-for-age-Z-score. OD: open defecation. MD: mean difference. PR: prevalence ratio.

Exposure	Outcome	Model	Point Estimate	CI Lower	CI Upper	Measure of Association	Subgroup
OD	HAZ	Unadjusted	-0.237	-0.279	-0.196	MD	Rural
OD	HAZ	Adjusted	0.011	-0.036	0.058	MD	Rural
OD	WHZ	Unadjusted	-0.168	-0.203	-0.134	MD	Rural
OD	WHZ	Adjusted	-0.028	-0.067	0.011	MD	Rural
OD	WAZ	Unadjusted	-0.254	-0.281	-0.228	MD	Rural
OD	WAZ	Adjusted	-0.009	-0.039	0.021	MD	Rural
OD	Stunting	Unadjusted	1.192	1.160	1.224	PR	Rural
OD	Stunting	Adjusted	1.006	0.975	1.038	PR	Rural
OD	Wasting	Unadjusted	1.151	1.109	1.194	PR	Rural
OD	Wasting	Adjusted	1.009	0.966	1.053	PR	Rural
OD	Underweight	Unadjusted	1.293	1.255	1.332	PR	Rural
OD	Underweight	Adjusted	1.013	0.978	1.049	PR	Rural
Shared	HAZ	Unadjusted	-0.063	-0.121	-0.004	MD	Rural
Shared	HAZ	Adjusted	0.026	-0.037	0.089	MD	Rural
Shared	WHZ	Unadjusted	-0.053	-0.104	-0.004	MD	Rural
Shared	WHZ	Adjusted	-0.027	-0.082	0.027	MD	Rural
Shared	WAZ	Unadjusted	-0.066	-0.104	-0.028	MD	Rural
Shared	WAZ	Adjusted	0.003	-0.037	0.044	MD	Rural
Shared	Stunting	Unadjusted	1.012	0.969	1.058	PR	Rural
Shared	Stunting	Adjusted	0.958	0.913	1.006	PR	Rural
Shared	Wasting	Unadjusted	1.008	0.981	1.072	PR	Rural
Shared	Wasting	Adjusted	0.981	0.916	1.049	PR	Rural
Shared	Underweight	Unadjusted	1.044	0.991	1.101	PR	Rural
Shared	Underweight	Adjusted	0.986	0.931	1.043	PR	Rural

Supplementary Table 6: Full Models for Urban Populations. HAZ: height-for-age-Z-score. WHZ: weight-for-height-Z-score. WAZ: weight-for-age-Z-score. OD: open defecation. MD: mean difference. PR: prevalence ratio.

Exposure	Outcome	Model	Point Estimate	CI Lower	CI Upper	Measure of Association	Subgroup
OD	HAZ	Unadjusted	-0.571	-0.712	-0.429	MD	Urban
OD	HAZ	Adjusted	-0.181	-0.347	-0.016	MD	Urban
OD	WHZ	Unadjusted	-0.191	-0.302	-0.079	MD	Urban
OD	WHZ	Adjusted	0.045	-0.088	0.178	MD	Urban
OD	WAZ	Unadjusted	-0.463	-0.553	-0.373	MD	Urban
OD	WAZ	Adjusted	-0.081	-0.183	0.021	MD	Urban
OD	Stunting	Unadjusted	1.502	1.376	1.641	PR	Urban
OD	Stunting	Adjusted	1.124	1.008	1.253	PR	Urban
OD	Wasting	Unadjusted	1.153	1.016	1.308	PR	Urban
OD	Wasting	Adjusted	0.954	0.818	1.113	PR	Urban
OD	Underweight	Unadjusted	1.588	1.437	1.755	PR	Urban
OD	Underweight	Adjusted	1.026	0.908	1.159	PR	Urban
Shared	HAZ	Unadjusted	-0.236	-0.336	-0.136	MD	Urban
Shared	HAZ	Adjusted	-0.061	-0.172	0.051	MD	Urban
Shared	WHZ	Unadjusted	-0.178	-0.260	-0.095	MD	Urban
Shared	WHZ	Adjusted	-0.056	-0.151	0.038	MD	Urban
Shared	WAZ	Unadjusted	-0.241	-0.306	-0.175	MD	Urban
Shared	WAZ	Adjusted	-0.053	-0.125	0.019	MD	Urban
Shared	Stunting	Unadjusted	1.175	1.086	1.270	PR	Urban
Shared	Stunting	Adjusted	1.041	0.954	1.138	PR	Urban
Shared	Wasting	Unadjusted	1.075	0.971	1.191	PR	Urban
Shared	Wasting	Adjusted	0.962	0.854	1.083	PR	Urban
Shared	Underweight	Unadjusted	1.253	1.143	1.373	PR	Urban
Shared	Underweight	Adjusted	1.025	0.924	1.137	PR	Urban

Supplementary Table 7: Rural Shared, Stratified by Number of Households Sharing. HAZ: height-for-age-Z-score. WHZ: weight-for-height-Z-score. WAZ: weight-for-age-Z-score. OD: open defecation. MD: mean difference. PR: prevalence ratio. "2 sharing", "3/4 sharing", and "5+ sharing" are all subgroups of the "any sharing" category and were analyzed in separate models.

Exposure	Outcome	Model	Point Estimate	CI Lower	CI Upper	Measure of Association	Subgroup
Shared	HAZ	Unadjusted	-0.095	-0.165	-0.024	MD	2 Sharing
Shared	HAZ	Adjusted	-0.026	-0.101	0.049	MD	2 Sharing
Shared	WHZ	Unadjusted	-0.061	-0.121	-0.001	MD	2 Sharing
Shared	WHZ	Adjusted	-0.022	-0.087	0.042	MD	2 Sharing
Shared	WAZ	Unadjusted	-0.091	-0.138	-0.044	MD	2 Sharing
Shared	WAZ	Adjusted	-0.024	-0.073	0.025	MD	2 Sharing
Shared	Stunting	Unadjusted	1.023	0.970	1.078	PR	2 Sharing
Shared	Stunting	Adjusted	0.983	0.928	1.041	PR	2 Sharing
Shared	Wasting	Unadjusted	1.003	0.931	1.081	PR	2 Sharing
Shared	Wasting	Adjusted	0.955	0.879	1.038	PR	2 Sharing
Shared	Underweight	Unadjusted	1.072	1.007	1.142	PR	2 Sharing
Shared	Underweight	Adjusted	1.012	0.946	1.083	PR	2 Sharing
Shared	HAZ	Unadjusted	-0.002	-0.107	0.103	MD	3/4 sharing
Shared	HAZ	Adjusted	0.122	0.009	0.236	MD	3/4 sharing
Shared	WHZ	Unadjusted	-0.032	-0.121	0.057	MD	3/4 sharing
Shared	WHZ	Adjusted	-0.024	-0.123	0.074	MD	3/4 sharing
Shared	WAZ	Unadjusted	-0.010	-0.078	0.057	MD	3/4 sharing
Shared	WAZ	Adjusted	0.068	-0.003	0.141	MD	3/4 sharing
Shared	Stunting	Unadjusted	0.980	0.903	1.063	PR	3/4 sharing
Shared	Stunting	Adjusted	0.902	0.822	0.989	PR	3/4 sharing
Shared	Wasting	Unadjusted	1.012	0.907	1.129	PR	3/4 sharing
Shared	Wasting	Adjusted	1.008	0.894	1.138	PR	3/4 sharing
Shared	Underweight	Unadjusted	0.961	0.870	1.061	PR	3/4 sharing
Shared	Underweight	Adjusted	0.893	0.801	0.996	PR	3/4 sharing
Shared	HAZ	Unadjusted	-0.007	-0.214	0.200	MD	5+ sharing
Shared	HAZ	Adjusted	0.135	-0.090	0.361	MD	5+ sharing
Shared	WHZ	Unadjusted	-0.055	-0.219	0.110	MD	5+ sharing
Shared	WHZ	Adjusted	-0.077	-0.262	0.108	MD	5+ sharing
Shared	WAZ	Unadjusted	-0.037	-0.171	0.097	MD	5+ sharing
Shared	WAZ	Adjusted	0.018	-0.127	0.164	MD	5+ sharing
Shared	Stunting	Unadjusted	1.025	0.886	1.187	PR	5+ sharing
Shared	Stunting	Adjusted	0.939	0.797	1.107	PR	5+ sharing
Shared	Wasting	Unadjusted	1.033	0.852	1.254	PR	5+ sharing
Shared	Wasting	Adjusted	1.097	0.898	1.341	PR	5+ sharing
Shared	Underweight	Unadjusted	1.091	0.920	1.292	PR	5+ sharing
Shared	Underweight	Adjusted	1.080	0.909	1.283	PR	5+ sharing

Supplementary Table 8: Urban Shared, Stratified by Number of Households Sharing. HAZ: height-for-age-Z-score. WHZ: weight-for-height-Z-score. WAZ: weight-for-age-Z-score. OD: open defecation. MD: mean difference. PR: prevalence ratio.

Exposure	Outcome	Model	Point Estimate	CI Lower	CI Upper	Measure of Association	Subgroup
Shared	HAZ	Unadjusted	-0.198	-0.333	-0.063	MD	2 Sharing
Shared	HAZ	Adjusted	-0.013	-0.163	0.137	MD	2 Sharing
Shared	WHZ	Unadjusted	-0.185	-0.299	-0.071	MD	2 Sharing
Shared	WHZ	Adjusted	-0.099	-0.227	0.028	MD	2 Sharing
Shared	WAZ	Unadjusted	-0.221	-0.311	-0.129	MD	2 Sharing
Shared	WAZ	Adjusted	-0.055	-0.153	0.042	MD	2 Sharing
Shared	Stunting	Unadjusted	1.111	0.995	1.238	PR	2 Sharing
Shared	Stunting	Adjusted	0.973	0.858	1.104	PR	2 Sharing
Shared	Wasting	Unadjusted	1.060	0.920	1.221	PR	2 Sharing
Shared	Wasting	Adjusted	0.959	0.816	1.127	PR	2 Sharing
Shared	Underweight	Unadjusted	1.242	1.095	1.410	PR	2 Sharing
Shared	Underweight	Adjusted	1.045	0.909	1.201	PR	2 Sharing
Shared	HAZ	Unadjusted	-0.216	-0.385	-0.047	MD	3/4 sharing
Shared	HAZ	Adjusted	-0.120	-0.300	0.059	MD	3/4 sharing
Shared	WHZ	Unadjusted	-0.161	-0.302	-0.019	MD	3/4 sharing
Shared	WHZ	Adjusted	-0.013	-0.168	0.142	MD	3/4 sharing
Shared	WAZ	Unadjusted	-0.221	-0.331	-0.112	MD	3/4 sharing
Shared	WAZ	Adjusted	-0.056	-0.173	0.062	MD	3/4 sharing
Shared	Stunting	Unadjusted	1.191	1.045	1.356	PR	3/4 sharing
Shared	Stunting	Adjusted	1.127	0.984	1.292	PR	3/4 sharing
Shared	Wasting	Unadjusted	1.069	0.894	1.279	PR	3/4 sharing
Shared	Wasting	Adjusted	0.946	0.775	1.155	PR	3/4 sharing
Shared	Underweight	Unadjusted	1.215	1.040	1.419	PR	3/4 sharing
Shared	Underweight	Adjusted	1.028	0.872	1.215	PR	3/4 sharing
Shared	HAZ	Unadjusted	-0.353	-0.569	-0.137	MD	5+ sharing
Shared	HAZ	Adjusted	-0.083	-0.315	0.147	MD	5+ sharing
Shared	WHZ	Unadjusted	-0.203	-0.369	-0.037	MD	5+ sharing
Shared	WHZ	Adjusted	-0.009	-0.198	0.181	MD	5+ sharing
Shared	WAZ	Unadjusted	-0.330	-0.464	-0.197	MD	5+ sharing
Shared	WAZ	Adjusted	-0.037	-0.179	0.104	MD	5+ sharing
Shared	Stunting	Unadjusted	1.289	1.107	1.502	PR	5+ sharing
Shared	Stunting	Adjusted	1.069	0.904	1.265	PR	5+ sharing
Shared	Wasting	Unadjusted	1.148	0.936	1.408	PR	5+ sharing
Shared	Wasting	Adjusted	0.963	0.759	1.223	PR	5+ sharing
Shared	Underweight	Unadjusted	1.365	1.145	1.628	PR	5+ sharing
Shared	Underweight	Adjusted	0.987	0.810	1.203	PR	5+ sharing
